# Association between genetic variants in hsa-miR-27a and hsa-miR-146a genes and male infertility

**DOI:** 10.5937/jomb0-50876

**Published:** 2024-11-16

**Authors:** Srećko Rajovski, Suzana Matijašević-Joković, Nikoleta Milanović, Nemanja Radovanović, Miloš Brkušanin, Dušanka Savić-Pavićević, Zorana Dobrijević, Goran Brajušković

**Affiliations:** 1 Private Healthcare Institution Clinical Hospital "Acibadem Sistina", Skopje, North Macedonia; 2 university of belgrade, faculty of biology, centre for human molecular genetic; 3 University of Belgrade, Institute for the Application of Nuclear Energy - INEP, Department for Metabolism, Belgrade

**Keywords:** male infertility, rs2910164, rs895819, polymorphism, miR-27a, miR-146a, muški sterilitet, rs2910164, rs895819, polimorfizam, miR-27a, miR-146a

## Abstract

**Background:**

miRNAs have enormous potential to be used as diagnostic and prognostic markers as well as therapeutic targets in male infertility and diseases of the reproductive system. This study aimed to investigate the association between the two functional genetic variants in the hsa-miR27a (rs2910164) and hsa-miR-146a gene (rs895819) and male infertility in North Macedonian population, as well as to test their association with the values of major seminal parameters.

**Methods:**

The case group included in this study comprised 158 men initially diagnosed with idiopathic male infertility. The control group included 126 age-matched healthy male volunteers who fathered at least one child.

**Results:**

We report the association of rs2910164 minor allele C for the first time with the increased susceptibility to asthenoteratozoospermia. Additionally, our results indicating the association of allele C with low sperm vitality are a novel finding. We did not demonstrate the association between genetic variant rs895819 and the risk of different types of male infertility. Still, the number of participants with CC genotype in subjects diagnosed with asthenoteratozoospermia was null, while in controls, it reached 7.2%. We further detected the rs895819 genotype-dependent difference in rapid progressive sperm motility.

**Conclusions:**

The association of rs2910164 and rs895819 with idiopathic male infertility in general is unlikely. However, both of these variants show an association with certain types of male infertility and with sperm abnormalities, which need to be confirmed in later studies in different ethnic groups.

## Abstract (sr)

**Uvod:**

miRNA molekule odlikuje izuzetan potencijal za upotrebu u vidu dijagnostičkih i prognostičkih biomarkera i terapijskih targeta kod muškog steriliteta i bolesti reproduktivnog sistema. Cilj ove studije je bilo ispitivanje asocijacije dveju funkcionalnih genetičkih varijanti gena hsa-miR-27a (rs2910164) i hsa-miR-146a (rs895819) i idiopatskog muškog steriliteta u populaciji Severne Makedonije i testiranje njihove asocijacije sa vrednostima seminalnih parametara.

**Metode:**

Grupu ispitanika činilo je 158 muškaraca sa inicijalnom dijagnozom idiopatskog muškog steriliteta. Kontrolna grupa je uključivala 126 muškaraca volontera podudarne starosti koji su imali barem jedno dete.

**Rezultati:**

Po prvi put je pokazana asocijacija manje učestalog alela C varijante rs2910164 sa povišenim rizikom za pojavu steriliteta kod muškaraca sa astenoteratozoospermijom. Takođe, rezultati koji ukazuju na asocijaciju alela C sa nižom stopom vijabilnosti spermatozoida predstavljaju novi nalaz. Nije pokazana asocijacija varijante rs895819 sa rizikom za pojavu muškog steriliteta. Ipak, procenat učesnika u studiji sa genotipom CC među muškarcima sa dijagnozom astenoteratozoospermije bio je 0, dok je u grupi kontrola iznosio 7,2%. Pored toga, ustanovljen je efekat varijante rs895819 na brzu progresivnu pokretljivost spermatozoida.

**Zaključak:**

Asocijacija rs2910164 i rs895819 sa idiopatskim muškim sterilitetom uopšteno nije potvrđena. S druge strane, za obe navedene varijante pokazana je asocijacija sa rizikom za pojavu određenih tipova muškog steriliteta, kao i sa abnormalnostima spermatozoida. Navedene rezultate je potrebno potvrditi u budućim studijama u drugim etničkim grupama.

## Introduction

Infertility is a disease of the male or female reproductive system defined by the failure to achieve a pregnancy after 12 months or more of regular unprotected sexual intercourse. Infertility impacts millions of people worldwide, often with devastating consequences [1].

MicroRNAs (miRNAs), an important class of non-coding RNAs (ncRNAs), are major regulators of gene expression and have been recognised as important regulators of various biological processes, including spermatogenesis. The presence of miRNAs in testis, epididymis, sperm cells, seminal plasma and extracellular vesicles (i.e., exosomes and microvesicles) and the known functions in the post-transcriptional regulation of gene expression associated with these molecules suggest that deregulation of their expression may lead to alterations in spermatogenesis and embryogenesis [2]. At the same time, miRNAs have enormous potential to be used as diagnostic and prognostic markers as well as therapeutic targets in male infertility and diseases of the reproductive system [3].

Numerous specific miRNAs have been discovered in spermatogonial stem cells (SSCs), with roles ranging from initiating or blocking the differentiation process to modulating the self-renewal mechanism. Hsa-miR-146a-5p (miR-146a) directly binds and inhibits a coregulator of retinoid receptors, known as the mediator complex subunit 1. High levels of retinoic acid were found to inhibit the expression of miR-146a in undifferentiated spermatogonia, while upregulation of miR-146a was found to be sufficient to antagonise the effects of retinoic acid on spermatogonia [4]. The genetic variant rs2910164 within miR-146a gene (GRCh38 5:160485411, C>G) is a functional polymorphism located in the »seed« region of the passenger strand and has been shown to generate a false C:U in the stem region, affecting the biogenesis of mature miR-146a [5]. Therefore, this genetic variant could influence the risk of developing male infertility through its complex effect on the intertwined processes of miR-146a biogenesis, mature strand selections and the function of the resulting mature miRNA, all potentially related to retinoic acid signalling. However, despite its experimentally well-supported functional properties, rs2910164 has never been evaluated as a male infertility-related genetic variant in a Slavic population.

Hsa-miR-27a-3p (miR-27a) is a member of the miR-27 family, which has two members (miR-27a and miR-27b) and has been identified as an oncogenic miRNA that plays an important role in cancer development [6]. This miRNA was predicted to target and regulate a specific H3K9 demethylase (lysine-specific demethylase 3A: KDM3A) with a proven role in male infertility in animal models. Disruption of epigenetic modification by KDM3A leads to a lack of spermatid chromatin after meiosis. Without the function of KDM3A in round spermatids, excessive methylation would lead to transcriptional suppression of transition protein 1 (TNP1) and protamine 1 (PRM1) genes and the occurrence of infertility in animal models. During the evolution of germline cells, epigenetic modifications via ncRNAs are crucial for regulating gene expression, genome stability and genome imprinting. Several studies have been conducted on human abnormalities and their association with miRNAs, including miR-27a. Norioun et al. [7] have shown that miR-27a is overexpressed in men with non-obstructive azoospermia. The genetic variant rs895819 (GRCh38 19:13836478, T>C) is located within a gene cluster encoding a single pri-miRNA for miR-23a, miR-24a and miR-27a. It was identified as an SNP that affects the sequence of the pre-miRNA terminal loop of miR-27a, leading to a change in its secondary structure and affecting the processing of miR-27a precursors [8]. Similarly, as for rs2910164, hypotheses on the potential involvement of rs895819 in the molecular pathogenesis of male infertility rely on its functional role in the biogenesis of mature miRNA and on the downstream effect of the disrupted miR-27a function on its target mRNAs indispensable for maintaining a normal spermatogenesis process.

Since current knowledge about the involvement of miRNA-related variants in the genetic basis of idiopathic male infertility is limited, the aim of this study was to investigate the association between the two functional genetic variants in the miR-27a (rs895819) and miR-146a genes (rs2910164) and male infertility in the North Macedonian population. Furthermore, we aimed at testing the association of these genetic variants with the values of major seminal parameters, which is even less investigated to date, and these results could provide valuable insight into the involvement of rs895819 and rs2910164, as well as miR-27a and miR-146a, in various aspects of male reproductive disorders.

## Materials and methods

The case group included in this study comprised 158 men initially diagnosed with idiopathic male infertility in 2018 at Acibadem Sistina Hospital, Skopje, North Macedonia. This research was conducted with the approval of the Ethics Committee of this medical institution (08-5110/01, May 2^nd^, 2018, Acibadem Sistina Hospital, Skopje, North Macedonia) and following the Helsinki Declaration of 1975. Written informed consent was obtained from all participants before their inclusion in the study. The control group included 126 healthy male volunteers from the North Macedonian population age-matched with the patients’ group who fathered at least one child.

Exclusion criteria for the case group were diagnosis of orchitis and obstruction of vas deferens. In all patients, cytogenetic analysis excluded chromosome abnormalities as a possible cause of infertility. Multiplex polymerase chain reaction (PCR) based screening detected no microdeletions of the azoospermia factor (AZF) region within the Y chromosome in the selected group. Semen analysis in infertile men was conducted according to World Health Organization (WHO) guidelines, and patients were stratified into subgroups based on the abnormalities in semen concentration, sperm motility or morphology. Patients’ basic data on age, serum testosterone levels and the results of semen analysis are described in detail in our previous publication [9] and are given in Table 1.

**Table 1:** Clinical characteristics of study participants diagnosed with idiopathic male infertility. Results are presented as mean±SD.<br>NOA – Nonobstructive azoospermia.

Clinical characteristic	NOA	Other types of infertility
N	14	144
Age (years)	35.57±5.91	39.79±8.30
Sperm parameters		
Ejaculate volume (mL)	4.06±2.04	3.02±1.55
Total sperm number (10^6^)	-	225.15±170.32
Sperm density (10^6^/mL)	-	75.34±52.16
Progressive motility (%)	-	33.57±13.87
Rapid progressive motility (%)	-	17.78±8.30
Average progression grade (0–4)	-	2.59±0.76
Vitality (%)	-	47.44±17.59

Peripheral blood samples from patients and healthy controls were collected into commercially available 3 ml tubes with ethylenediaminetetraacetic acid (EDTA). Genomic DNA was isolated from peripheral blood samples using magnetic bead technology following the manufacturer’s protocol. Genotyping was performed by using TaqMan™ SNP Genotyping Assays (C___3056952_20 and C__15946974_10) (Applied Biosystems, Waltham, MA, USA), according to the manufacturer’s protocol. Real-time PCR amplification was carried out by Step One Real-time PCR System (Applied Biosystems, Waltham, MA, USA).

The exact test implemented in SNPStats software (Institut Català d’Oncologia, Barcelona, Spain) was used to assess the potential deviations of genotype distributions from Hardy-Weinberg equilibrium (HWE). The same software was used to test the associations of rs2910164 and rs895819 with the risk of idiopathic male infertility and with sperm viability assessed according to the threshold of 60% recommended by WHO. Odds ratios (ORs) with the corresponding 95% confidence intervals (CIs) were used as effect size measures in association tests, while *P* values <0.05 were considered to be statistically significant. The best-fitting association model among the five tested (log-additive, codominant, dominant, recessive and overdominant), was determined using the Akaike information criterion (AIC). By employing AIC as an estimator of the relative quality of statistical models, the best-fitting model was selected among the alternative models according to the lowest corresponding value of AIC. For quantitative results of semen analysis, the Kolmogorov-Smirnov test was used to assess the normality of the distribution of results, while the F-test was employed to analyse the equality of variances in study groups. Two-tailed Student’s T-test was used to compare normally distributed results, while in cases of significant deviations from the normal distribution, the Mann–Whitney U test was applied.

## Results

Table 1 summarises the basic data and semen parameters of patients diagnosed with male infertility. Most of the infertile men engaged in the present study were diagnosed with infertility types other than NOA (91%), including oligozoospermia, oligoasthenozoospermia, oligoteratozoospermia, oligoasthenoteratozoospermia, asthenoteratozoospermia and teratozoospermia. The values of semen parameters demonstrated high dispersion concerning the mean due to variable results associated with different diagnoses and characteristics of different forms of infertility. The average age of healthy fertile controls (40.85±10.58, not shown in Table 1) at sampling was almost matched to the age of infertile individuals.

Genotyping of rs895819 and rs2910164 was successful for 99.4% (157/158) of study participants diagnosed with idiopathic infertility and 98.4% (125/127) of fertile controls. Genotype distributions, summarised in Table 2, were not found to significantly deviate from HWE in control subjects (*P*=1 and *P*=0.82 for rs895819 and rs2910164, respectively).

**Table 2:** Association of genetic variants rs895819 and rs2910164 with male infertility. Abbreviations: OR – odds ratio; CI – confidence interval; AIC – Akaike information criteria. ^a^Adjusted for age.

SNP	Genetic model	No of controls<br>(%)	No of cases<br>(%)	cases vs controls		
				OR (95% CI) ^a^	*P* value ^a^	AIC
**rs895819**						
	Codominant					
	TT	68 (54.4)	76 (48.4)	1.00	0.58	393
	TC	48 (38.4)	68 (43.3)	1.29 (0.79–2.12)		
	CC	9 (7.2)	13 (8.3)	1.26 (0.51–3.14)		
	Dominant					
	TT	68 (54.4)	76 (48.4)	1.00	0.3	391
	TC+CC	57 (45.6)	81 (51.6)	1.29 (0.80–2.06)		
	Recessive					
	TT+TC	116 (92.8)	144 (91.7)	1.00	0.79	392
	CC	9 (7.2)	13 (8.3)	1.13 (0.46–2.74)		
	Overdominant					
	TT+CC	77 (61.6)	89 (56.7)	1.00	0.36	391.2
	TC	48 (38.4)	68 (43.3)	1.25 (0.77–2.03)		
	Log-additive					
	-	-		1.20 (0.82–1.74)	0.35	391.2
**rs2910164**						
	Codominant					
	GG	69 (55.2)	84 (53.5)	1.00	0.58	393
	GC	47 (37.6)	67 (42.7)	1.29 (0.79–2.12)		
	CC	9 (7.2)	6 (3.8)	1.26 (0.51–3.14)		
	Dominant					
	GG	69 (55.2)	84 (53.5)	1.00	0.3	391
	GC+CC	56 (44.8)	73 (46.5)	1.29 (0.80–2.06)		
	Recessive					
	GG+GC	116 (92.8)	151 (96.2)	1.00	0.79	392
	CC	9 (7.2)	6 (3.8)	1.13 (0.46–2.74)		
	Overdominant					
	GG+CC	78 (62.4)	90 (57.3)	1.00	0.36	391.2
	GC	47 (37.6)	67 (42.7)	1.25 (0.77–2.03)		
	Log-additive					
	-	-	-	1.20 (0.82–1.74)	0.35	391.2

According to the results of genetic association tests presented in Table II, neither rs895819 norrs2910164 demonstrated an association with the risk of idiopathic infertility in our North Macedonian male cohort. The results for all genetic models tested remained statistically insignificant for comparing genotype distributions between infertile men and age-matched fertile controls (Table 2).

When we stratified infertile subjects into groups according to the results of semen analysis in terms of abnormalities in semen parameters and the related diagnosis, infertile men with asthenoteratozoospermia showed a higher frequency of rs2910164 heterozygous genotype compared to controls (Table 3). According to AIC score, the over-dominant genetic model was selected as the best-fitting model of association between rs2910164 and asthenoteratozoospermia, while the corresponding OR value for GC vs. homozygous genotypes was 3.66 (95%CI 1.38– 9.71, *P* = 0.0068) (Table 3), indicative of an increased risk of asthenoteratozoospermia associated with GC genotype. Additionally, statistically significant differences in genotype distributions between infertile men diagnosed with asthenoteratozoospermia and fertile controls was demonstrated for dominant and codominant genetic models (*P* = 0.013 and *P* = 0.025, respectively), with an increased risk of asthenoteratozoospermia associated with GC genotype or with a combination of genotypes which includes GC. For rs895819, only a statistical trend of significance (0.05 P<0.1) was reached for a recessive genetic model of association with asthenoteratozoospermia. However, none of the subjects with asthenoteratozoospermia had rs895819 genotype CC, while the frequency of this genotype in the control group was 7.2% (Table 3).

**Table 3:** Comparison of genotype distributions of rs895819 and rs2910164 between subjects with asthenoteratozoospermia and fertile controls. Abbreviations: OR – odds ratio; CI – confidence interval; AIC – Akaike information criteria. ^a^ adjusted for age;^ b^ statistical trend of significance; ^*^Statistically significant results are shown in bold.

SNP	Genetic model	No of controls<br>(%)	No of cases<br>(%)	cases vs controls		
				OR (95% CI) ^a^	P value ^a^	AIC
**rs895819**						
	Codominant					
	TT	68 (54.4)	10 (45.5)	1.00	0.11	127.7
	TC	48 (38.4)	12 (54.5)	1.72 (0.68–4.32)		
	CC	9 (7.2)	0 (0)	0.00 (0.00–NA)		
	Dominant					
	TT	68 (54.4)	10 (45.5)	1.00	0.44	129.5
	TC+CC	57 (45.6)	12 (54.5)	1.43 (0.58–3.56)		
	Recessive					
	TT+TC	116 (92.8)	22 (100)	1.00	0.082^b^	127.1
	CC	9 (7.2)	0 (0)	0.00 (0.00–NA)		
	Overdominant					
	TT+CC	77 (61.6)	10 (45.5)	1.00	0.16	128.1
	TC	48 (38.4)	12 (54.5)	1.94 (0.77–4.87)		
	Log-additive					
	-	-		1.05 (0.50–2.19)	0.9	130.1
**rs2910164**						
	Codominant					
	GG	69 (55.2)	6 (27.3)	1.00	**0.025**	124.7
	GC	47 (37.6)	15 (68.2)	3.77 (1.35–10.51)*		
	CC	9 (7.2)	1 (4.5)	1.27 (0.14–11.80)		
	Dominant					
	GG	69 (55.2)	6 (27.3)	1.00	**0.013**	123.9
	GC+CC	56 (44.8)	16 (72.7)	**3.35 (1.22–9.16)**		
	Recessive					
	GG+GC	116 (92.8)	21 (95.5)	1.00	0.63	129.8
	CC	9 (7.2)	1 (4.5)	0.61 (0.07–5.08)		
	Overdominant					
	GG+CC	78 (62.4)	7 (31.8)	1.00	**0.0068**	122.7
	GC	47 (37.6)	15 (68.2)	**3.66 (1.38–9.71)**		
	Log-additive					
	-	-	-	1.87 (0.93–3.78)	0.082b	127.1

Both analysed variants showed no statistically significant differences in genotype distributions between subjects with other types of idiopathic male infertility and fertile controls (results not shown). We considered it statistically unjustified to compare genotype frequencies between patients diagnosed with non-obstructive azoospermia (NOA) and the control group since the number of participants in this group of infertile men was just 14 in our study cohort (Table 1).

Non-azoospermic infertile patients with rs2910164 GC genotype presented with lower sperm viability, as defined by the threshold of 60% viable sperm (Table 4). Similarly, as for association with asthenoteratozoospermia, the over-dominant genetic model was selected as the best-fitting model of association between rs2910164 and low sperm viability and the calculated OR for GC vs. homozygous genotypes was 2.65 (95%CI 1.12–6.24, P=0.02) (Table 4), suggesting that GC genotype associates with lower sperm viability. Although the AIC score was the lowest for the over-dominant model, statistical significance was reached for the other two genetic association models – dominant and log-additive (*P*=0.023 and *P*=0.039, respectively). For all the tested genetic models for which the statistical significance was reached, as well as for the comparison of GC vs GG under the codominant model, the obtained ORs for GC genotypes and the genotype combinations that incorporate heterozygotes were over 2, indicating an increase in risk for lower sperm viability associated with tested compared to referent genotypes.

**Table 4:** Comparison of genotype distributions of rs895819 and rs2910164 between infertile subjects with sperm vitality of at least 60% and those with a lower percentage of live spermatozoa. Abbreviations: OR – odds ratio; CI – confidence interval; AIC – Akaike information criteria. ^a^ adjusted for age<br>^b^ statistical trend of significance<br>^*^ Statistically significant results are shown in bold.

SNP	Genetic model	Vit. 60% (%)	Vit. <60% (%)	Vit. <60% vs Vit. 60%		
				OR (95% CI) ^a^	P value ^a^	AIC
**rs895819**						
	Codominant					
	TT	16 (47.1)	47 (46.1)	1.00	0.63	160
	TC	14 (41.2)	48 (47.1)	1.17 (0.51–2.65)		
	CC	4 (11.8)	7 (6.9)	0.59 (0.15–2.29)		
	Dominant					
	TT	16 (47.1)	47 (46.1)	1.00	0.93	158.9
	TC+CC	18 (52.9)	55 (53.9)	1.04 (0.48–2.26)		
	Recessive					
	TT+TC	30 (88.2)	95 (93.1)	1.00	0.37	158.1
	CC	4 (11.8)	7 (6.9)	0.55 (0.15–2.00)		
	Overdominant					
	TT+CC	20 (58.8)	54 (52.9)	1.00	0.55	158.5
	TC	14 (41.2)	48 (47.1)	1.27 (0.58–2.79)		
	Log-additive					
	-	-		0.90 (0.49–1.67)	0.75	158.8
**rs2910164**						
	Codominant					
	GG	24 (70.6)	50 (48.5)	1.00	0.066 ^b^	156
	GC	9 (26.5)	50 (48.5)	2.69 (1.13–6.37)*		
	CC	1 (2.9)	3 (2.9)	1.39 (0.14–14.20)		
	Dominant					
	GG	24 (70.6)	50 (48.5)	1.00	**0.023**	154.3
	GC+CC	10 (29.4)	53 (51.5)	**2.56 (1.11–5.88)**		
	Recessive					
	GG+GC	33 (97.1)	100 (97.1)	1.00	0.98	159.5
	CC	1 (2.9)	3 (2.9)	0.97 (0.10–9.69)		
	Overdominant					
	GG+CC	25 (73.5)	53 (51.5)	1.00	**0.02**	154.1
	GC	9 (26.5)	50 (48.5)	**2.65 (1.12–6.24)**		
	Log-additive					
	-	-	-	**2.19 (1.01–4.75)**	**0.039**	155.2

**Figure 1:**
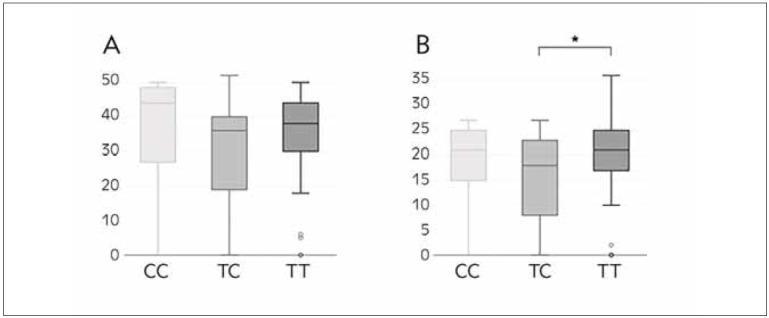
Sperm progressive motility (a) and rapid progressive motility (b) in infertile men across genotypes of rs895819. Data represent the median and interquartile range (IQR). P<0.05 is indicated by an asterisk.

Even though rs895819 showed no evidence of association with male infertility diagnosis, as well as with progressive motility score (Figure 1A), carriers of TC genotype displayed a lower percentage of sperm with rapid progressive motility compared to infertile subjects with the most common TT genotype (*P*=0.018) (Figure 1B).

## Discussion

Male infertility, which is a major contributing factor in around 40–50% of cases of a couple’s inability to conceive, is a multifactorial disorder with a recognised causative role of genetic factors involved in its complex molecular basis [10] [11]. Besides chromosome aberrations and single gene mutations with high penetrability, which account for a relatively small percentage of infertile cases, genetic variants with modest or low penetrability were identified as contributors to genetic susceptibility to develop male infertility [11]. These risk-associated genetic variants were discovered through large genome-wide association studies (GWAS), high-throughput techniques, and candidate gene-based approaches [11]. For such genetic association studies focused on single genes or groups of functionally related genes, candidates were selected based on their involvement in the molecular basis of spermatogenesis, sperm functionality, oxidative stress response and other major processes related to infertility. In the recent decade, miRNA molecules emerged as important regulators of the spermatogenesis process, while their dysregulation was associated with a reduced sperm count, lower sperm viability and/or motility, as well as abnormalities in sperm morphology [4] [12]. Therefore, miRNA-related genes seem plausible candidates for genetic association studies on male infertility.

MiRNA variants commonly selected for case-control studies are predicted or experimentally confirmed as functional polymorphisms that affect multiple aspects of miRNA-based mechanisms. This includes miRNA biogenesis, their localisation, sequence of the mature miRNA molecules, strand selection, efficiency of post-transcriptional regulation of targeted genes, and the selection of mRNA targets [13]. Given that these genetic variants could simultaneously affect multiple processes regulated by the same miRNA, most of them were evaluated in the context of various human pathological conditions, including malignant and immunological diseases, metabolic disorders, and other common health issues [14] [15] [16] [17] [18]. Still, data on the effect of commonly evaluated genetic variants on the susceptibility to male infertility are scarce or even lacking for certain aspects or types of infertility-related abnormalities.

We selected genetic variants within genes encoding miR-146a and miR-27a for our present study. Both of these miRNA molecules are expressed in germ cells and were previously found to have an altered expression level in biological samples obtained from infertile men compared to healthy fertile controls [4] [7] [19]. Additionally, their role in regulating key cellular processes disrupted in disorders of sperm maturation was revealed, while several studies confirmed their involvement in oxidative stress response and/or inflammation [2] [4] [7] [20] [21] [22]. It is worth noting that miR-27a is an androgen-regulated miRNA that stimulates androgen signalisation via a positive feedback loop [23]. We aimed to evaluate the effects of variants with functional properties, so we selected an extensively analysed genetic variant rs2910164 within the *hsa-miR-146a* gene. This genetic variant exhibits it functional effect on miR-146a function by altering the structure of the pre-miRNA hairpin, which affects the efficiency of mature miRNA biogenesis. Furthermore, rs2910164 could potentially affect the leader strand selection and change the sequence of the seed region of miR-146a-3p, which has consequences on the selection of the target gene pool [24]. The second selected genetic variant is located within a host gene of a cluster of miRNAs miR-23a, miR-24-2 and miR-27a. This genetic variant alters the sequence of the terminal loop of pre-miR-27a and potentially affects the biogenesis of mature miR-27a by influencing the processing of primary miRNA by the Drosha enzyme [25] [26].

To date, rs2910164 was evaluated as a susceptibility variant in male infertility in two studies conducted in the Turkish population [27] and in Han Chinese [28]. No evidence of such genetic association was found in both of these studies. However, a study by Tunçdemir et al. [27] evaluated the association of rs2910164 with idiopathic oligospermia without considering other types of infertility. Furthermore, their publication did not include results on the potential association between rs2910164 genotypes and the values of semen parameters, which we intended to evaluate in the present study. Lu et al. [28], on the other hand, evaluated their large study case-control study on the association of rs2910164 with idiopathic male infertility in general and with specific types of infertility diagnosed according to the results of semen analysis. Still, their criteria for patient selection differed from ours since their case group included men with normospermia, who, in fact, represented the largest percentage of their infertile subjects [28].

Our study was the first one to analyse the effects of rs2910164 on a Slavic population with a European Caucasian genetic background. This genetic variant shows a significant global variation in allele frequencies. For this reason, we expected that our novel findings could differ from the previous and would be valuable for a more precise evaluation of the effects of rs2910164 in the context of the potential influence of ethnicity as a confounder. Regarding the results obtained for the association between this genetic variant and overall idiopathic male infertility, our findings match the previously published reports from Turkish and Han Chinese populations since our results did not support the supposed association [27] [28]. However, these two studies did not evaluate the association of rs2910164 with asthenoteratozoospermia and provided no results on the potential effects of this genetic variant on abnormal sperm morphology or the combination of sperm motility and abnormal phenotype [27] [28]. Therefore, our results suggest that the association of rs2910164 minor allele C with increased susceptibility to asthenoteratozoospermia could not be compared with any previous findings. Additionally, our results indicating the association of allele C with low sperm vitality are a novel finding (Table 4).

When it comes to rs895819 in a gene encoding miR-27a, a single previous association study related to male infertility was conducted in a Serbian cohort of infertile men [29]. This previous case-control evaluation in a genetically close related population to the present one reported a lack of statistically significant association between rs895819 and idiopathic male infertility risk, consistent with the study’s finding in the North Macedonian cohort. However, the number of infertile men and fertile controls in this previous study was relatively low, which warranted additional evaluation of the effects of rs895819 [29]. Our present study did not demonstrate the association between this genetic variant and the risk of different types of male infertility. Still, the number of participants with CC genotype in a small group of subjects diagnosed with asthenoteratozoospermia was null, while in controls, it reached 7.2%. Therefore, it is possible that the association of rs895819 under a recessive or allelic genetic model would be detected in a larger validation study. Our novel finding of the genotype-dependent difference in rapid progressive sperm motility in infertile men should also be evaluated in future more extensive studies. However, our finding is consistent with a previous report on the effect of miR-27a expression on sperm progressive motility [20].

A relatively small number of participants in both case and control groups is the main limitation of this study. Therefore, in order to provide a more precise estimation and to make further conclusions about the potential association between the analysed genetic variants and male infertility, including the effect on sperm parameters, the study size needs to be enlarged. The association of rs2910164 and rs895819 with idiopathic male infertility is generally unlikely, considering the matching results between the present and previous analyses. However, both of these variants show an association with certain types of male infertility and with sperm abnormalities, which need to be confirmed in later studies in different ethnic groups.

## Dodatak

### Acknowledgements

The research was financially supported by the Ministry of Science, Technological Development and Innovation of the Republic of Serbia (Agreement no. 451-03-47/2023-01/200042). We owe gratitude to the Diagnostic laboratories and Assisted Reproduction (IVF) Department of Clinical Hospital »Acibadem Sistina« employees for their assistance in collecting and preparing samples.

### Conflict of interest statement

All the authors declare that they have no conflict of interest in this work.

### List of abbreviations

AIC, Akaike information criterion; 

AZF, azoospermia factor; 

CI, confidence interval; 

EDTA, ethylenedi-aminetetraacetic acid; 

GWAS, Genome-Wide Association Studies; 

HWE, Hardy-Weinberg equilibrium; 

IQR, interquartile range; 

KDM3A, lysine-specific demethylase 3A; 

miR-146a, hsa-miR-146a-5p; 

miR-27a, Hsa-miR-27a-3p; 

miRNAs, microRNAs; 

ncRNAs, non-coding RNAs; 

NOA, non-obstructive azoospermia; 

OR, odds ratio; 

PCR, polymerase chain reaction; 

PRM1, protamine 1; 

SSCs, spermatogonial stem cells; 

TNP1, transition protein 1; 

WHO, World Health Organization.
